# Bloodstream Infections caused by *Klebsiella pneumoniae and Serratia marcescens* isolates co-harboring NDM-1 and KPC-2

**DOI:** 10.1186/s12941-021-00464-5

**Published:** 2021-08-30

**Authors:** Taniela Bes, Debora Nagano, Roberta Martins, Ana Paula Marchi, Lauro Perdigão-Neto, Hermes Higashino, Gladys Prado, Thais Guimaraes, Anna S. Levin, Silvia Costa

**Affiliations:** 1 Infectious Diseases Division, Hospital das Clínicas da Universidade de São Paulo, São Paulo, Brazil; 2 Institute of Tropical Medicine of the University of São Paulo, Avenida Dr. Enéas Carvalho de Aguiar, 470; LIM 49, São Paulo, CEP 05403-000 Brazil; 3 Infection Control, Hospital das Clínicas Faculdade de Medicina da Universidade de Sao Paulo, Sao Paulo, Brazil

**Keywords:** *Enterobacteriaceae*, Carbapenem resistance, Carbapenemases, *bla*_KPC-2_, *bla*_NDM-1_

## Abstract

Carbapenem-resistant *Enterobacteriaceae* are a worldwide health problem and isolates carrying both *bla*_KPC-2_ and *bla*_NDM-1_ are unusual. Here we describe the microbiological and clinical characteristics of five cases of bloodstream infections (BSI) caused by carbapenem-resistant *Klebsiella pneumoniae* and *Serratia marcescens* having both *bla*_KPC-2_ and *bla*_NDM-1_. Of the five blood samples, three are from hematopoietic stem cell transplantation patients, one from a renal transplant patient, and one from a surgical patient. All patients lived in low-income neighbourhoods and had no travel history. Despite antibiotic treatment, four out of five patients died. The phenotypic susceptibility assays showed that meropenem with the addition of either EDTA, phenylboronic acid (PBA), or both, increased the zone of inhibition in comparison to meropenem alone. Molecular tests showed the presence of *bla*_KPC-2_ and *bla*_NDM-1_ genes. *K. pneumoniae* isolates were assigned to ST258 or ST340 by whole genome sequencing. This case-series showed a high mortality among patients with BSI caused by *Enterobacteriae* harbouring both carbapenemases. The detection of carbapenemase-producing isolates carrying both *bla*_KPC-2_ and *bla*_NDM-1_ remains a challenge when using only phenotypic assays. Microbiology laboratories must be alert for *K. pneumoniae* isolates producing both KPC-2 and NDM-1.

## Introduction

In the last decade, several studies have reported the emergence of Gram-negative bacteria carrying multiple carbapenemases isolated from patients with distinct pathologies [1–6]. However, clinical isolates harbouring simultaneously KPC-2 and NDM-1 carbapenemases are less common [7–12]. Previous reports describing the production of both carbapenemases include clinical isolates of *Klebsiella pneumoniae* [7–9], *Klebsiella oxytoca* [10], *Enterobacter cloacae* [11, 12], and *Enterobacter hormaechei* [13], collected from rectal swabs, blood samples, urinary tract infections, and wound infections as well. Regarding *Serratia marcescens*, clinical isolates harbouring simultaneously KPC-2 and IMP-10 [14] or KPC-2 and SRT-2 [15] were described.

The production of carbapenemases, such as *Klebsiella pneumoniae* carbapenemase (KPC) and the New Delhi metallo-β-lactamase (NDM), constitutes one of the most important mechanisms of resistance to β-lactam antibiotics [16]. As the emergence of carbapenem-resistant clinical isolates has become a serious clinical challenge due to the limited treatment options, the presence of multiple carbapenemases from the same strain further aggravates this issue.

Regarding the Brazilian data, NDM producers were originally detected in the southern regions of Brazil and have since moved into other states [17]. According to surveillance data, bacterial pathogens producing KPC alone are the main cause of bloodstream infections (BSI) in intensive care unit patients (21%) in the state of São Paulo [18]. Reports from Brazil also indicate that NDM and KPC producers carry resistance genes other than KPC-2 and NDM-1 conferring resistance to other antimicrobials classes rather than β-lactam [12, 13, 19]. The present study describes the clinical and microbiological features of five, namely *K. pneumoniae* (n = 4) and *S. marcescens* (n = 1), co-harboring KPC-2 and NDM-1, collected from bloodstream infections (BSI).

## Material and methods

Four carbapenem-resistant *K. pneumoniae* and one carbapenem-resistant *S. marcescens* were obtained from blood cultures during 2012 and 2016. The patients were hospitalized at Hospital das Clínicas, Faculdade de Medicina da Universidade de São Paulo, a tertiary teaching hospital in São Paulo, Brazil. These isolates belong to the Microbiology Laboratory Biobank.

Vitek II (bioMerieux, Marcy-l'Étoile, France) was used to identify the isolates KP1411, KP4301, KP158, and SM1581. The isolate KP4990 was identified using matrix-assisted laser desorption ionization-time of flight mass spectrometry (MALDI-TOF MS; Bruker, Billerica, Massachusetts, USA).

To confirm the susceptibility pattern, the minimum inhibitory concentrations (MICs) were determined using Sensititre Gram Negative GNX3F AST Plate (TREK Diagnostic Systems, Cleveland, OH, USA). The antimicrobials tested were aztreonam, meropenem, imipenem, ceftazidime, colistin, amikacin, tigecycline, and ciprofloxacin [20]. The susceptibility test results were interpreted according to Clinical Laboratory Standards Institute (CLSI) M100 [21] criteria. Isolates were considered susceptible if the MIC of carbapenems were ≤ 1 mg/mL, intermediate if MIC = 2 mg/mL, and resistant with MICs ≥ 4 mg/mL [22]. *Escherichia coli* ATCC25922 was used as a quality control strain and *K. pneumoniae* ATC 700603 as carbapenem-resistant *Enterobacteriaceae* control strain.

To precisely detect the genes coding for the carbapenemases KPC and NDM, we performed PCR using previously described primers for *bla*_KPC-2_ and *bla*_NDM-1_ [22–24]. The amplicons were submitted to Sanger Sequencing using MegaBACE 1000 (ABI 3730 DNA Analyser; Applied Biosystems, Alameda, CA) to confirm gene identity.

To determine the genotypic profile including resistance and virulence genes, whole genome sequencing (WGS) was performed by Illumina MiSeq. For WGS, total DNA was extracted with Illustra bacteria genomicPrep Mini Spin Kit (GE Healthcare Life Sciences, Marlborough, USA). DNA quality was verified using the NanoDrop spectrophotometer (Thermo Scientific, Delaware, USA). The quality of the files generated in the sequencing was evaluated by FastQC v.0.11.3 and Trimmomatic v.0:33. Genome assembly was performed using Velvet Optimiser v.2.2.5 and annotated with Prokka v.1:11 [25–27]. The sequence type (ST) of the isolates was determined with MLSTfinder tool [28] and confirmed in the PubMLST (https://pubmlst.org/kpneumoniae/info/primers.shtml) database. The gene *bla*_KPC-2_ was manually investigated using Artemis v.16.0.0.

Additional phenotypic analysis was done to confirm carbapenem resistance, using the disk diffusion (DD) method described by Migliavacca et al. [29], and the CLSI breakpoints for carbapenems. Meropenem (MPM) commercial disks containing 10 µg (Sensidisc DME, Araçatuba, BRA) were added with 0.05 M of ethylenediaminetetraacetic acid (EDTA) (Sigma-Aldrich, St. Louis, USA) and/or 20 μg/mL of phenylboronic acid (PBA) (Sigma-Aldrich, St. Louis, USA). According to Migliavacca et al. [29], a difference of ≥ 4 mm in the inhibition diameter zone was used as criteria to determine whether the isolates produce a serine carbapenemase, a metallo-β-lactamase, or both, in presence of EDTA and/or PBA, respectively, when compared to the MPM disk alone. *E. coli* ATCC25922 was used as negative quality control.

## Results

**Fig. 1:**
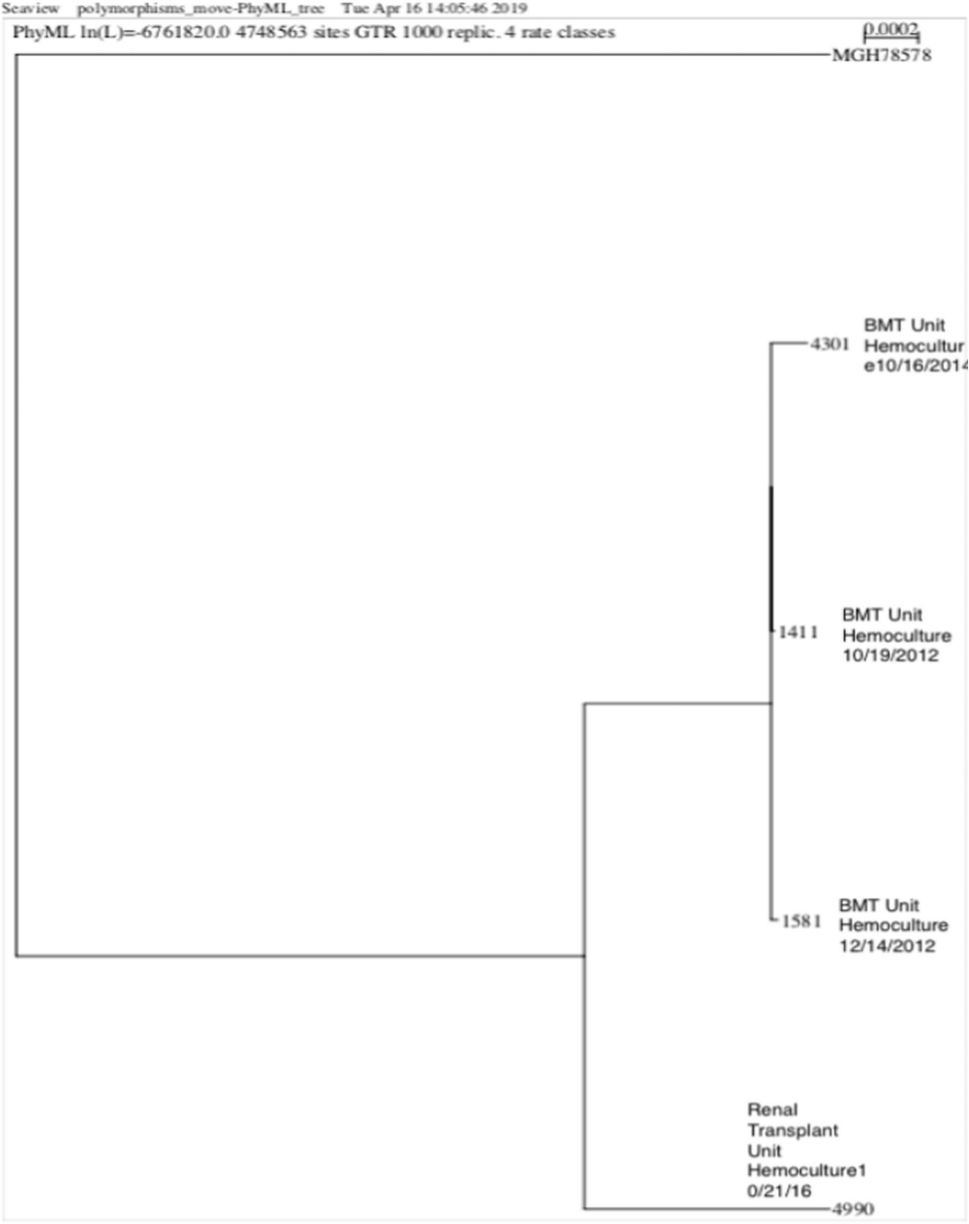
Phylogenies of *K. pneumoniae* showing the same clone ST340 in the BMT unit and the globally disseminated, ST258 in Renal Transplant Unit

The five clinical isolates analysed in the present study were collected from BSI of five patients submitted to hematopoietic stem cell transplant (n = 3), renal transplant (n = 1), and soft tissue surgery (n = 1) (see Fig. 1). All patients lived in low-income neighbourhoods in São Paulo, had less than eight years of schooling, and had no history of international travel.

The patients in this study were individuals with severe conditions and prolonged hospitalization, varying between 16 to 51 days after diagnosed with BSI by multi-drug resistant (MDR) agents. Three of the four patients infected by *K. pneumoniae* died within the course of the treatment, two of them deceased two days after the diagnosis, and one died 40 days after BSI culture turned positive. The patient infected by *S. marcescens* died 11 days after diagnosis.

After blood culture results, tigecycline and colistin were added to the treatment of *the K. pneumoniae* (n = 4) infected patients, who at that point had been receiving meropenem or cefepime. The patient infected with *S. marcescens* was receiving ciprofloxacin and clindamycin, increased to tigecycline and fosfomycin after the blood culture result. Detailed clinical data is shown in the Table 1.

**Table 1:** Underlying clinical conditions, antibiotic therapy, outcome, and phenotypic analyze

Isolate ID Year MLST	Underlying clinical condition	Impiric treatment	Culture guided added tratment	Outcome	Phenotypic Tests—Serina-β-lactamases (DD mm)	Phenotypic Tests—MBLs (DD mm)	Carbapenem-bac
*K. pneumoniae* (KP1411) 10/19/2012 ST340	**Myelodysplastic syndrome** Leukocytes: 100 Neutrophils: 0 Allogeneic BMT (10/11/2012)	Meropenem	Tigecycline Colistin	Death 2 days after blood positive culture	**DD (mm)** IMP: 0 ERT: 0 MPM: 0 **DD (mm) agent’s combination:** MPM + PBA: 14 mm MPM + EDTA + PBA: 17 mm	**DD (mm)** IMP: 0 ERT: 0 MPM: 0**DD (mm) agent’s combination:** MPM + EDTA: 0 (phantom zone 14) MPM + EDTA + PBA: 17 mm	Positive
*K. pneumoniae* (KP1581) 12/14/2012 ST340	**Severe aplastic anemia** Leukocytes: 100 Neutrophils: 0 Not related allogeneic TCTH (12/09/2012)	Meropenem	Tigecycline Colistin	Death 2 days after blood positive culture	**DD (mm)** IMP: 23 mm ERT: 16 mm MPM: 20 mm **DD (mm) agent’s combination:** MPM + PBA: 23 mm MPM + EDTA + PBA: 23 mm	**DD (mm)** IMP: 23 mm ERT: 16 mm MPM: 20 mm **DD (mm) agent’s combination:** MPM + EDTA: 21 mm MPM + EDTA + PBA: 23 mm	Positive
*K. pneumoniae* (KP4301) 10/16/2014 ST340	**Non-Hodgkin lymphoma** **Peripheral T-cell lymphoma** Leukocytes: 3,33 Neutrophils: 2,43 Autologous BMT 9/17/2014	Cefepime	Tigecycline Colistin	Discharge from hospital 23 days after blood positive culture, for a total of 64 days hospitalization	**DD (mm)** IMP: 0 ERT: 0 MPM: 0 **DD (mm) agent’s combination:** MPM + PBA: 13 mm MPM + EDTA + PBA: 18 mm	**DD (mm)** IMP: 0 ERT: 0 MPM: 0 **DD (mm) agent’s combination:** MPM + EDTA: 10 (phantom zone 14) MPM + EDTA + PBA: 18 mm	Positive
*S. marcescens* (SM1756) 6/14/2013	**Necrotizing fasciitis** Amputation of the right arm (05/31/13) Complication: Ischemic cecum perforation (6/07/2013)	Ciprofloxacin Clindamycin	Tigecycline Fosfomycin	Death 11 days after blood positive culture	**DD (mm)** IMP: 13 mm ERT: 17 mm MPM: 23 mm **DD (mm) agent’s combination:** MPM + PBA: 25 mm MPM + EDTA + PBA: 26 mm	**DD (mm)** IMP: 13 mm ERT: 17 mm MPM: 23 mm **DD (mm) agent’s combination:** MPM + EDTA: 19 mm MPM + EDTA + PBA: 26 mm	Positive
*K. pneumoniae* (KP4990) 10/21/16 ST258	**Kidney transplant due Chronic Kidney Disease** (10/13/16) Urinary tract infection (10/20/16) Graft Nephrectomy (11/23/16)	Meropenem	Ceftazidime + Avibactam	Death 40 days after blood positive culture	**DD (mm)** IMP: 6 mm ERT: 0 MPM: 0 (phantom zone 7 mm) **DD (mm) agent’s combination:** MPM + PBA: 14 mm MPM + EDTA + PBA: 16 mm (phantom zone 20)	**DD (mm)** IMP: 6 mm ERT: 0 MPM: 0 (phantom zone 7 mm) **DD (mm) agent’s combination:** MPM + EDTA: 9 mm (phantom zone 20) MPM + EDTA + PBA: 16 mm (phantom zone 20)	Positive

*MPM* Meropenem, *IMP* Imipenem, *ERT* Ertapenem, *PBA* Phenylboronic Acid, *DD* Diffusion Disk, *MLST* Multilocus sequence typing

Microdilution susceptibility testing demonstrated that all isolates were resistant to at least two carbapenems. All the isolates were susceptible to ceftazidime plus avibactam and tigecycline. However, the use of ceftazidime with avibactam was not available in Brazil for clinical use during the study and just one patient was competent to receive the drug under a research protocol. The isolates KP1411, KP4301, and KP4990 were susceptible to colistin, being KP1411 also susceptible to amikacin (Table 1). Phenotypic tests with specific β-lactamase inhibitors revealed the presence of serine carbapenemases and metallo-β-lactamases. Meropenem with either EDTA and/or PBA showed an increased zone of inhibition in comparison to meropenem disk alone.

The isolates KP1411, KP4301, and KP4990 had full growth around the meropenem disk and, when either EDTA and/or PBA was added to the meropenem disk, an inhibition zone higher than 9 mm was obtained. However, the isolates KP1411 and KP4301 presented a double ring reaction surrounding the disk with MPM and EDTA, two clear different zones of growth for the same bacteria. The experiment was repeated three times, all with the same pattern. The isolate KP4301 presented a phantom zone around the disk with MPM, EDTA, and PBA. The remaining two isolates, *S. marcescens* SM1581 and KP1581 did not show differences higher than 4 mm in the zone of inhibition in any of the combinations (Table 1).

In addition to *bla*_KPC-2_ in all the isolates, the WGS analysis led to the identification of other resistance and virulence genes, such as *fos*A in the isolate KP4301. The *bla*_NDM-1_ gene was detected only by PCR and confirmed by DNA sequencing by Sanger, but not in WGS, which could be due to loss of gene due our extraction protocol which was designed for genomic DNA and could not categorize low copy plasmid DNA or impairment of sequencing platform. Besides the resistance genes, the WGS analysis showed virulence genes related with adhesion, efflux pumps, iron acquisition, regulation, and secretion systems in the four *K. pneumoniae* isolates. Each of the genes were analyzed separately (Table 2). The WGS data were used for further MLST analysis of the isolates representing the two common STs for *K. pneumoniae*, ST258 and ST340. Isolates KP1411, KP4301, and KP1581 were assigned to ST340 and isolate KP4990 to ST258.

**Table 2:** Minimal inhibitory concentration, virulence and resistance-associated genes identified by whole genome sequence

Isolate Year MLST	Beta-Lactams’ resistance genes	Aztreonam (MIC)	Meropenem (MIC)	Imipenem (MIC)	Ceftazidime (MIC)	DD Ceftazidime Avibactam	Aminoglycosides resistance genes	Amikacin (MIC)	Colistin (MIC)	Tigecycline (MIC)	Quinolone resistance genes	Ciprofloxacin (MIC)	Virulence genes adherence	Virulence gene efflux pump	Virulence gene iron acquisition
*K. pneumoniae (1411) 10/19/2012 ST340*	blaCTX-M-15 blaKPC-2 blaSHV-11 blaTEM-1B	≥ 32 [R]	≥ 16 [R]	≥ 16 [R]	≥ 16 [R]	25 mm [S]	aac (3)-Iia aac(6′)Ib-cr aadA2 strA strB	< 4 [S]	4	0.5	aac (6')Ib-cr oqxA oqxB QnrB1	≥ 4 [R]	mrkA/B/C/D/F/H/I/J filmA/B/C/D/E/F/G/H/I/K	acrA/B	iutA entA/B/C/D/E/F/S fepA/B/C/D/G iroE/N
*K. pneumoniae (1581) 12/14/2012 ST340*	blaKPC-2 blaSHV-11	≥ 32 [R]	4 [R]	2	16 [R]	24 mm [S]	aph (3′)-Ia aadA2	8	≥ 4 [R]	0.5	oqxA oqxB	≥ 4 [R]	mrkC/H/I/J filmA/B/D/E/G/H/I	acrA/B	entA/B/C fepB/G iroN
*K. pneumoniae (4301) 10/16/2014 ST340*	blaKPC-2 blaSHV-5 blaSRT-2	≥ 32 [R]	≥ 16 [R]	≥ 16 [R]	≥ 16 [R]	30 mm [S]	Aph(3′)-Via	16	0,5	0.5	OqxA oqxB fosA dfrA14	≥ 4 [R]	mrkA/B/C/D/F/H/I/J filmA/B/C/D/E/F/G/H/I/K	acrA/B	iutA entA/B/C/D/E/F/S fepA/B/C/D/G iroE/N
*S. marcescens (1756) 6/14/2013*	blaKPC-2 blaSHV-5 blaSRT-2	≥ 32 [R]	4 [R]	8	≥ 16 [R]	24 mm [S]	aacA4 aac(6′)-Ic aadB	16	≥ 4 [R]	2	aac(6′)-Ib-cr	≥ 4 [R]	shlA/B rssA/B		
*K. pneumoniae (4990) 10/21/16 ST258*	blaCTX-M-14 blaKPC-2 blaSHV-11 blaTEM-1B	≥ 32 [R]	≥ 64[R]	≥ 16 [R]	≥ 16 [R]	23 mm [S]	rmtB aac(3)-Iid aph(3′)-Ia aadA2 aph(6)-Id aph(3′′)-Ib	≥ 64 [R]	2	0.5	oqxA oqxB	≥ 4 [R]	mrkA/B/C/D/F/H/I/J filmA/B/C/D/E/F/G/H/I/K	acrA/B	iutA entA/B/C/D/E/F/S fepA/B/C/D/G iroE/N

*MLST* Multilocus sequence typing, *R* Resistant, *S* Susceptible, *MIC* Minimum inhibitory concentrations, *DD* Diffusion Disc

Due to the phenotypic profile of the samples, PCR analysis was performed and detected *bla*_KPC-2_ and *bla*_NDM-1_ genes in all five strains. The PCR result was confirmed by Sanger sequencing.

## Discussion

To the best of our knowledge, this is the first report describing *K. pneumoniae* and *S. marcescens* clinical isolates harbouring simultaneously *bla*_KPC-2_ and *bla*_NDM-1_. The presence of these resistance genes was not previously described in *K. pneumoniae* ST258 and ST340 worldwide.

The ST258 emerged during the early 2000s as a hybrid clone created by recombination between ST11, ST442, and the ST340, which is a single-locus variant of ST11 [30]. These isolates tend to be pan-resistant, restricting the therapeutic options, particularly to *S. marcescens* isolates, since they are intrinsic resistance to polymyxins [31].

The dissemination of NDM-1 and KPC-2 in Brazil is of great concern since a *Providencia rettgeri* isolate carrying NDM-1 was described in the South region in 2013 [19]. Subsequently, Rozales et al. [17] published a study analysing 1134 isolates of *Enterobacteriaceae*, among which 11 isolates (0.97%) harboured *bla*_NDM-1_. Noteworthy, none of these cases had a history of traveling outside Brazil, which suggest local acquisition. The isolates analysed in the present study were collected from patients with risk factors contributing to infection by multidrug resistant pathogens, e.g., low socioeconomic class, low educational background, severe clinical conditions, and poor functional status [8, 31, 32].

Outside the hospital setting, another aggravating factor is the lack of basic sanitation in the country. Ecological surveillance studies found KPC-2 producing *K. pneumoniae* isolates (ST340) in urban rivers in the city of São Paulo, Brazil [33]. Such finding may confirm improper treatment of hospital sewage being discarded in the rivers and rising a hypothesis regarding the correlation among environmental and hospital isolates.

Our isolates displayed high-level of resistance to β-lactams, aminoglycosides, and fluoroquinolones. Carbapenem-resistant enterobacteria isolates carrying *bla*_NDM-1_ are more likely to be resistant to several antibiotics. They are often accompanied by other resistance genes, associated with resistance to β-lactams, fluoroquinolones, and aminoglycosides, that corroborates Kumarasamy et al., 2010 data, who analysed 111 isolates of *K. pneumoniae* producing NDM-1 from India and United Kingdom. Their isolates displayed resistance to fluoroquinolones and aminoglycosides [7].

Although the isolate KP1581 is colistin-resistant, the WGS did not show plasmid mediated resistance genes or previously described chromosomal mutations that lead to colistin resistance. Therefore, we speculate that the resistance derived from virulence genes encoded from lipopolysaccharide (LPS) *rfb* locus. The genes *wzm* and *wzt,* codify a transmembrane ATP-binding cassette transporter (ABC). It plays an important role in the synthesis of cell surface LPS [34]. The Wzt protein dictates the specificity of the substrate and the glycan chain length, which serves as an export signal recognized by the ABC transporter. The isolate KP1581 carries just the *wzm* gene. Thus, our hypothesis is that it has *wzt* trapped by the transporter in a state incapable to complete the O-PS export. It might explain the colistin resistance, specifically because, in *K. pneumoniae*, the cytosolic glycan synthesis and export are obligatorily coupled [35].

The main limitations of this study are the small number of samples and the fact they belong to different genera. Even though, the presence of *bla*_KPC-2_ and *bla*_NDM-1_ might have contributed to an unfavourable clinical outcome, considering that four of the patients died from the same conditions without blood culture clearance.

In conclusion, the detection of carbapenemase-producing isolates carrying both *bla*_KPC-2_ and *bla*_NDM-1_ remains a challenge. Additional effort is required to identify isolates when using only phenotypic assays. Thus, routine microbiology laboratories must be on alert for isolates possessing both β-lactamases.

## Data Availability

The isolates’ WGS are kept at the GeneBank under the numbers: KP1411-*Klebsiella pneumoniae*—QOIJ00000000. KP1581-*Klebsiella pneumoniae*—QOII00000000. KP4301-*Klebsiella pneumoniae*—JABBZC000000000. SM1756-*Serratia marcescens*—QJPQ00000000. KP4990-*Klebsiella pneumoniae*—QOTZ00000000. The isolates’ Sanger sequence are kept at the GeneBank under the numbers: (Gene blaKPC e blaNDM). blaNDM_1411-MT721962. blaNDM_4990-MT721963. blaNDM_1756-MT721964. blaNDM_4301-MT721965. blaNDM_1581-MT721966. blaKPC_4990-MT721967. blaKPC_4301-MT721968. blaKPC_1581-MT721969. blaKPC_1411-MT721970. blaKPC_1756-MT665969.
